# What we can learn from Studies out of China

**DOI:** 10.1093/geroni/igaf122.1607

**Published:** 2025-12-31

## Abstract

This session includes five diverse studies out of China addressing life expectancy, exercise prescriptions, smoking and depression, cardiovascular risk in HIV and psychological resilience and cognitive frailty.

